# Metropolitan Hospital Sunday Fund

**Published:** 1905-01-14

**Authors:** 


### METROPOLITAN HOSPITAL SUNDAY FUND.

### MR. GEORGE HERRING UNANIMOUSLY ELECTED.

A meeting of the Council of the Metropolitan Hospital Sunday Fund was held on Tuesday (10th inst.) at the Mansion House, the Lord Mayor presiding. The business before the Council was the election of the Committee and other honorary officers, and general business. Letters of regret had been received from Sir Sydney Waterlow, the Bishops of Stepney and Islington, the Archdeacon of London, Prebendary Ridgeway, and others who were unable to be present. The Secretary, Sir Edmund Hay Currie, said the only vacancies to be filled were two on the Committee of Distribution. These were caused by the much regretted death of Mr. Herman Hoskier, an active member since 1888, and the resignation, through ill health, of another old member, Captain James Candy. It was proposed to fill the first vacancy by the election of Mr. Felix Schuster, Chairman of the Union and Smiths Bank, and a colleague of the late Mr. Hoskier. For the second, he desired to submit the name of a gentleman who, more than anyone else, had helped the Fund during the last five years, and whose munificent donations had amounted to over £50,000, nearly £12,000 having been contributed by him last year. The request made year by year since Mr. Herring's connection with the Fund, that he would consent to identify himself still more intimately with it by serving on one of the committees, had again been renewed, and he was glad to be in a position to say that Mr. Herring had now given his consent, provided that it were the wish of the Council that he should take office. Both gentlemen were unanimously elected, and the members of the General Purposes Committee, as well as the honorary officers, were re-elected for the ensuing year. Sir Edmund Hay Currie remarked that he would be glad if it were possible for any two members of the Council, who, with the secretary, constituted the special committee for dealing with surgical appliance orders and hospital letters, to attend at the office in the mornings, when the press of work was great, several hours having to be devoted to correspondence with some 2,000 clergy.

